# The neural underpinnings of intergroup social cognition: an fMRI meta-analysis

**DOI:** 10.1093/scan/nsab034

**Published:** 2021-03-24

**Authors:** Carrington C Merritt, Jennifer K MacCormack, Andrea G Stein, Kristen A Lindquist, Keely A Muscatell

**Affiliations:** Department of Psychology and Neuroscience, University of North Carolina at Chapel Hill, Chapel Hill, NC 27599, USA; Department of Psychology and Neuroscience, University of North Carolina at Chapel Hill, Chapel Hill, NC 27599, USA; Department of Psychiatry, University of Pittsburgh, Pittsburgh, PA 15213, USA; Department of Psychology, University of Wisconsin–Madison, Madison, WI 53705, USA; Department of Psychology and Neuroscience, University of North Carolina at Chapel Hill, Chapel Hill, NC 27599, USA; Department of Psychology and Neuroscience, University of North Carolina at Chapel Hill, Chapel Hill, NC 27599, USA; Lineberger Comprehensive Cancer Center, University of North Carolina at Chapel Hill, Chapel Hill, NC 27515, USA; Carolina Population Center, University of North Carolina at Chapel Hill, Chapel Hill, NC 27515, USA

**Keywords:** intergroup bias, social cognition, fMRI, meta-analysis, race

## Abstract

Roughly 20 years of functional magnetic resonance imaging (fMRI) studies have investigated the neural correlates underlying engagement in social cognition (e.g. empathy and emotion perception) about targets spanning various social categories (e.g. race and gender). Yet, findings from individual studies remain mixed. In the present quantitative functional neuroimaging meta-analysis, we summarized across 50 fMRI studies of social cognition to identify consistent differences in neural activation as a function of whether the target of social cognition was an in-group or out-group member. We investigated if such differences varied according to a specific social category (i.e. race) and specific social cognitive processes (i.e. empathy and emotion perception). We found that social cognition about in-group members was more reliably related to activity in brain regions associated with mentalizing (e.g. dorsomedial prefrontal cortex), whereas social cognition about out-group members was more reliably related to activity in regions associated with exogenous attention and salience (e.g. anterior insula). These findings replicated for studies specifically focused on the social category of race, and we further found intergroup differences in neural activation during empathy and emotion perception tasks. These results help shed light on the neural mechanisms underlying social cognition across group lines.

## Introduction

From an early age, humans tend to categorize ourselves and others as ‘us versus them’ (Mahajan and Wynn, 2012; Liberman *et al.*, 2017). These categorizations can lead individuals to enact disparate behaviors toward in-group and out-group members. For example, individuals tend to behave in ways that favor in-group members (i.e. in-group favoritism) and disfavor out-group members (Tajfel and Turner, 1979; Tajfel, 1982; Balliet *et al.*, 2014). Such is the case when White individuals assign less harsh legal punishments to White (*vs* Black) targets (Johnson *et al.*, 2002) or grant more comprehensive medical care to White patients compared to patients of other races (Drwecki *et al.*, 2011; Kaseweter *et al.*, 2012). Further, perceptions of out-groups as more homogenous than one’s in-group (i.e. out-group homogeneity effect) can also influence social behavior in intergroup interactions (Judd and Park, 1988; Ostrom and Sedikides, 1992; Brauer, 2001; Hughes *et al.*, 2019). This can manifest in the individuals’ tendency to be less discerning in their perception of emotional expressions of out-group members (Richeson *et al.*, 2007), which may engender discriminatory behavior via stereotyping and prejudice (Hughes *et al.*, 2019).

It is clear from this behavioral literature that social categorizations matter: the ways in which we think about one another vary depending on perceived in-group *vs* out-group status (Brewer, 2007). Further, these differences in in-group *vs* out-group social cognition can underlie biased social behaviors (Brewer, 1999; Major *et al.*, 2013; Molenberghs and Louis, 2018). However, it is less clear ‘how’ exactly an individual’s group membership sets into action the neural processes that may ultimately mediate biased behavior.

### Neuroscience of intergroup social cognition

Neuroimaging approaches have been widely used over the past two decades to address this ‘how’ by examining the neural mechanisms that underlie social cognitive processes directed toward in-group *vs* out-group members. For instance, consistent with the in-group favoritism effect, functional magnetic resonance imaging (fMRI) data reveal greater activity in the ventral striatum for in-group members (Telzer *et al.*, 2015) and more amygdala activity for out-group members (Cunningham *et al.*, 2004; Hein *et al.*, 2010), suggesting that in-group members may be perceived as more valuable and/or rewarding and out-group members might be more uncertain, ambiguous or aversive. Moreover, consistent with an out-group homogeneity effect, greater activity in the dorsomedial prefrontal cortex (dmPFC) for in-group members (Adams *et al.*, 2010; Mathur *et al.*, 2010) and less activity in dmPFC for out-group members (Harris and Fiske, 2006) further underscore the fact that people may more likely attribute unique and rich mental qualities to in-group compared to out-group members.

However, inconsistencies in the literature also abound, making it difficult to draw definitive conclusions about the neural mechanisms underlying intergroup social cognition. For instance, some neuroimaging studies reveal greater insula and dorsal anterior cingulate cortex (dACC) activation to out-group members during emotion perception tasks (Liu *et al.*, 2015; Watson and de Gelder, 2017), whereas others show greater activity in these regions during in-group emotion perception (Lee *et al.*, 2008; Xu *et al.*, 2009; Azevedo *et al.*, 2013; Cikara and Van Bavel, 2014). Similarly, some studies in which group membership is based on race find greater amygdala activation in response to ‘out-group’ faces, which may reflect other-race negativity bias (Hart *et al.*, 2000; Phelps *et al.*, 2000; Cunningham *et al.*, 2004). However, neuroimaging studies involving social categorization based on minimal groups (e.g. red team *vs* blue team) have demonstrated greater amygdala activation in response to ‘in-group’ members (Van Bavel *et al.*, 2008). These results suggest that the specific type of social grouping under consideration (i.e. race *vs* minimal group) may influence the neural regions engaged during social cognition across group lines (Van Bavel *et al.*, 2008; Cikara and Van Bavel, 2014). These inconsistencies are further complicated by the fact that individual neuroimaging studies are more prone to Type I errors due to small sample sizes and insufficient statistical corrections (Wager *et al.*, 2007).

Since past studies in this area assess diverse social categories (e.g. race and minimal groups) and social cognitive processes (e.g. empathy and emotion perception), it is important to identify the core neural mechanisms underlying in-group and out-group social cognition across the literature. This is particularly important in light of the fact that it is not possible to make inferences about generalized intergroup neural processes from single studies that only investigate one type of social group (e.g. race). Further, while some studies offer evidence of the neural regions involved in generalized social categorization (Cikara *et al.*, 2017; Lau and Cikara, 2017), still relatively little is known about how the brain distinguishes between ‘us’ and ‘them’ more broadly. Meta-analysis is useful in this context because it allows us to identify the most reliable patterns of activation across several studies, regardless of the social category of distinction in any individual study. Further, this analytic tool overcomes the limitations associated with sample size, power and experimental design inherent in individual fMRI studies (Cremers *et al.*, 2017; Turner *et al.*, 2018) to help reveal the functional neuroanatomy or ‘neural reference space’ consistently related to a process of interest (i.e. intergroup social cognition; Lindquist *et al.*, 2012).

Additionally, research needs to address how neural activity in intergroup contexts varies according to both the ‘social category’ assessed and ‘social cognitive process’ involved. Thus, we also aimed to use meta-analysis to identify how the neural mechanisms of intergroup social cognition may reliably vary as a function of a specific ‘social category’ (i.e. race) and two particular ‘social cognitive processes’ (i.e. empathy and emotion perception). We focused on ‘race’ as a key social category, given the importance of race-based bias in inter-race contexts (Richeson *et al.*, 2007; Han, 2018) and the consequences of these behaviors on the health and well-being of marginalized racial group members (Major *et al.*, 2013). Further, we focused on ‘empathy’ and ‘emotion perception’, given that these are two of the most studied processes in the intergroup social cognition fMRI literature (Molenberghs and Louis, 2018), and it is commonly argued that these social cognitive processes allow perceivers to represent the uniquely human experiences of group members that are important to intergroup relations (Richeson *et al.*, 2007; Zaki and Cikara, 2015). Investigating these in-group/out-group differences in the neural underpinnings of social cognition according to racial grouping and among the specific social cognitive processes of empathy and emotion perception will provide a more nuanced understanding of how group membership may shape behavior in intergroup contexts, especially in the case of race-based biases in social behavior.

### The present study

In sum, this meta-analysis addressed four primary questions: (i) Are a core set of brain regions reliably involved during social cognition across various ‘social categories’ and ‘social cognitive processes’? (ii) Do the neural correlates of in-group/out-group social cognition consistently differ when ‘race’ is the category on which the target’s group membership is based? (iii) Does neural activation across in-group *vs* out-group consistently differ when ‘empathy’ and ‘emotion perception’ are the specific social cognitive processes engaged? (iv) Finally, within the specific social category of race, does neural activation consistently differ according to the specific social cognitive process engaged (i.e. empathy and emotion perception)?

Our analysis expands upon a prior meta-analysis (Shkurko, 2013) of ∼30 studies which found that the amygdala, ACC, fusiform gyrus and right insula were reliably involved in distinguishing between in-group and out-group members generally. The current meta-analysis contains a total of 50 studies published through 2000–2018 and utilizes multilevel kernel density analysis (MKDA) as opposed to activation likelihood estimation technique used in Shkurko (2013). Moreover, the present paper extends this prior work, which did not distinguish between a variety of social categories and types of social cognition, to examine the more specific neural correlates of intergroup social cognition for the social category of race and the specific social cognitive processes of empathy and emotion perception.

## Methods

### Study selection and search strategy

Following Preferred Reporting Items for Systematic Reviews and Meta-Analyses (PRISMA) standards (Liberati *et al.*, 2009), our search strategy first collected relevant papers from PubMed and PsycINFO. We searched for English-language publications of fMRI studies that examined processing of in-group/out-group human targets. The initial search terms used were ‘fMRI + in-group + out-group’ and ‘fMRI + group membership’. We also used these terms in conjunction with various social categories to capture as many different in-group/out-groups as possible (see Supplemental Materials).

**Fig. 1.**
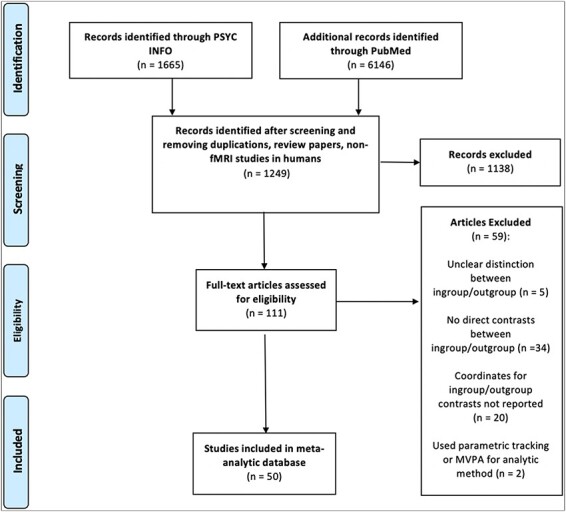
PRISMA diagram summarizing the literature search and study screening, eligibility and inclusion process.

Titles and abstracts of papers from these searches were reviewed to eliminate any clearly irrelevant studies or duplicates. The initial searches also resulted in several narrative review papers, which we mined for additional papers but excluded from the database of studies. Next, we completed full-text screening to eliminate studies that did not meet the following criteria: (i) participants were healthy, non-medicated adults; (ii) used fMRI to measure BOLD signal as an index of neural activity; (iii) coordinates of activation for contrasts were reported in either Montreal Neurological Institute (MNI) or Talairach space and (iv) reported contrasts that directly compared processing of distinguishable in-group *vs* out-group (or vice versa) targets. We included both contrasts involving explicit processing of in-group/out-group distinctions (e.g. categorization of stimuli by group membership) and contrasts involving implicit processing of these distinctions (i.e. passive viewing of stimuli representing group membership). Coordinates for both region-of-interest and whole-brain analyses were included, consistent with prior MKDA approaches (Kober and Wager, 2010; Lindquist *et al.*, 2012, 2016; MacCormack *et al.*, 2020). These inclusion criteria resulted in a total of 50 papers in the final database, which together yielded 116 contrasts. See Figure 1 for PRISMA diagram and Supplementary Table S2 for characteristics of the included studies.

### Data collection

Data extraction was completed by two coders (i.e. the first and third authors), with each coder reviewing all articles separately. Thus, all studies were double-coded and cross-checked to identify discrepancies. If discrepancies were noted, both coders reviewed the article again to determine the accurate data to report. Each article was coded for the following elements: sample size, social cognitive process, stimuli (e.g. still face images and videos), social group (e.g. race, culture, gender and minimal), relational status of the target and reference (i.e. in-group or out-group), and coordinates of peak activation.

## Data analysis

MKDA (see Supplemental Materials for more information) was implemented through the Matlab toolbox NeuroElf (http://neuroelf.net/). Consistent with MKDA and neuroimaging meta-analytic procedures (Wager *et al.*, 2007; Lindquist *et al.*, 2016; Van Hoorn *et al.*, 2019), contrast coordinates in Talairach space were first converted to MNI space and then convolved using a smoothing kernel of 12 mm, ultimately producing binary indicator contrast maps. Weights were placed on each study based on the square root of the sample size and whether the study used fixed or random effects. Fixed-effect studies were down-weighted by 0.75 to reduce the influence of those studies. By weighting studies in this manner, MKDA allows for higher-quality (i.e. higher powered and more generalizable) studies to have greater impact on the meta-analytic results (Kober and Wager, 2010). The weighted averages of the kernels across individual study contrasts were used to produce contrast maps based on the proportion of activation near a given voxel from *N* contrasts. This proportion is thresholded by comparing it to a null distribution created through Monte Carlo simulations (5000 samples) that compute the likelihood of finding any activation in any voxel within gray matter (excluding white matter). For all analyses, we set this a priori threshold to a stringent height-based threshold of *p* < 0.001 (family-wise error-corrected for multiple comparisons) to determine whether voxels were significant. Results thus represent the neural regions displaying the most consistent activation for a given contrast (i.e. ‘in-group > out-group’) when averaged across all studies.

First, we investigated the neural reference space of brain regions consistently activated during ‘in-group > out-group’ and ‘out-group > in-group’ contrasts across all study-level contrasts. Identifying these neural reference spaces allowed us to determine the core set of brain regions consistently associated with in-group *vs* out-group social cognition across the literature, regardless of the social cognitive process or group category studied. To supplement these primary contrasts, we also conducted meta-analytic contrasts in which we contrasted both of the aforementioned sets of contrasts against each other as follows: [(‘in-group > out-group’) > (‘out-group > in-group’)] and [(‘ > in-group) > (in-group > out-group’)]. These meta-analytic contrasts allowed us to determine which clusters of activation were relatively more consistent for ‘in-group > out-group’ contrasts relative to ‘out-group > in-group’ contrasts and vice versa.

Second, we examined the neural correlates of social cognition specifically for contrasts in which race was the social category of distinction. To do so, we investigated the neural reference space for each ‘racial in-group > racial out-group’ and ‘racial out-group > racial in-group’ contrast. Again, we supplemented these primary contrasts with meta-analytic contrasts, [(‘racial in-group > racial out-group’) > (‘racial out-group > racial in-group’)], to determine the relative specificity of activation for each contrast.

Third, we examined how consistent differences in neural activation might differ based on the specific social cognitive process engaged. Thus, we investigated the neural reference space for ‘in-group > out-group’ and ‘out-group > in-group’ by specific social cognitive process. We focus in the main text on empathy and emotion perception, given their prevalence in the literature and importance for predicting biases in behavior (Richeson *et al.*, 2007; Zaki and Cikara, 2015; Molenberghs and Louis, 2018). Results for other social cognitive processes are presented in Supplementary Table S3.

Finally, we examined the neural reference spaces for specific social cognitive processes (i.e. empathy and emotion perception) specifically within race-based contrasts. Results for other types of social cognition within race-specific contrasts are presented in Supplementary Table S4.

## Results

### Overall differences in functional activation for in-group *vs* out-group

We first identified the neural reference space of regions more consistently activated for ‘in-group > out-group’, irrespective of task or social group (515/520 points; 115/116 contrasts). This analysis revealed consistent activity in the bilateral anterior insula, including a left anterior insula cluster (−36, 15, 10; *k* = 260) that extended into the claustrum and a right anterior insula cluster (43, 20, 8; *k* = 260) that extended into the right inferior frontal gyrus (iFG) and right precentral gyrus. A third cluster was centered in the right dmPFC (8, 47, 27; *k* = 260), extending into the superior frontal gyrus.

**Fig. 2.**
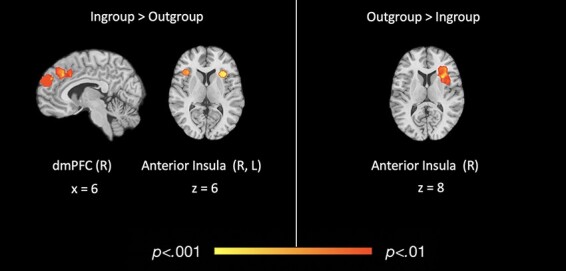
Regions of significant, consistent functional activation for overall in-group *vs* out-group social cognitive processing.

Next, we examined the neural reference space of regions more consistently activated for ‘out-group > in-group’, irrespective of task or social group (515/520 points; 115/116 contrasts). Here, we observed one significant cluster of activity, with its peak in the right anterior insula (33, 12, 13; *k* = 3060), extending into the right iFG and precentral gyrus. Thus, both the ‘in-group > out-group’ and ‘out-group > in-group’ contrasts revealed similar, but distinct, peaks in the anterior insula (see Table 1 and Figure 2).

**Table 1.** Coordinates for overall differences in functional activation for in-group *vs* out-group

Region	Brodmann	*x*	*y*	*z*	*k*	Max	Mean
**Overall in-group > out-group**							
*LH anterior insula (cluster)*	13	−36	15	10	260	0.33	0.25
LH anterior insula	13	−36	15	10	^a^	0.33	0.27
LH claustrum	n/a	−23	20	6	^a^	0.25	0.22
*RH anterior insula (cluster)*	13	43	20	8	260	0.26	0.21
RH inferior frontal gyrus	13	43	20	8	^b^	0.26	0.21
RH precentral gyrus	44	51	19	8	^b^	0.21	0.21
*RH dorsomedial PFC (cluster)*	9	8	47	27	260	0.28	0.18
RH dorsomedial PFC	9	8	47	27	^c^	0.28	0.17
RH dorsomedial PFC	9	4	57	26	^c^	0.20	0.18
**Overall out-group > in-group**							
*RH anterior insula (cluster)*	13	33	12	13	306	0.21	0.10
RH anterior insula	13	33	12	13	^d^	0.21	0.11
RH inferior frontal gyrus	13	40	26	10	^d^	0.17	0.10
RH anterior insula	13	35	2	11	^d^	0.14	0.10
RH precentral gyrus	44	47	4	11	^d^	0.12	0.09

*Notes*: Brodmann = Brodmann area; *x*, *y*, *z* = coordinates in MNI space; *k* = cluster size in mm^3^; max = maximum value within a cluster; mean = average value within a cluster; LH = left hemisphere; RH = right hemisphere. All analyses were *k*-threshold corrected at *P* < 0.001

a Associated subclusters of the LH anterior insula.

b Associated subclusters of the RH inferior frontal gyrus.

c Associated subclusters of the RH dorsomedial PFC.

d Associated subclusters of the RH anterior insula.

### Meta-analytic contrasts for in-group *vs* out-group

**Fig. 3.**
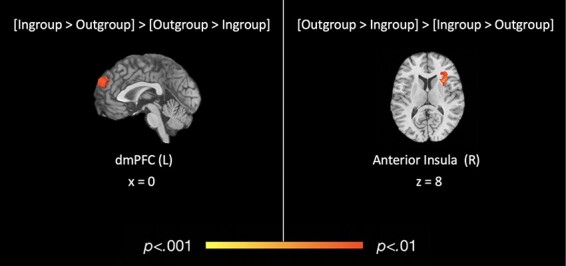
Regions of significant, consistent functional activation for meta-analytic contrasts of in-group *vs* out-group social cognitive processing.

We also conducted meta-analytic contrasts [(‘in-group > out-group’) > (‘out-group > in-group’)] and [(‘out-group > in-group’) > (‘in-group > out-group’)] to determine which regions, if any, were more consistently active for ‘in-group > out-group’ relative to ‘out-group > in-group’ and vice versa. The [(‘in-group > out-group’) > (‘out-group > in-group’)] contrast revealed a significant cluster in the left dmPFC (0, 51, 36; *k* = 100), while the [(‘out-group > in-group’) > (‘in-group > out-group’)] contrast revealed a significant cluster of activation in the right anterior insula (33, 12, 13; *k* = 130) extending into the right iFG and precentral gyrus (see Table 2 and Figure 3).

**Table 2.** Coordinates for meta-analytics contrasts for overall in-group *vs* out-group

Region	Brodmann	*x*	*y*	*z*	*k*	Max	Mean
**(In > out) > (out > in)**							
**LH dorsomedial PFC**	9	0	49	32	100	0.11	0.09
LH dorsomedial PFC	9	0	49	32	^a^	0.11	0.09
LH dorsomedial PFC	10	0	60	28	^a^	0.10	0.09
**(Out > in) > (in > out)**							
*RH anterior insula (cluster)*	13	33	12	13	130	0.21	0.10
RH anterior insula	13	33	12	13	^b^	0.21	0.10
RH anterior insula	13	35	2	11	^b^	0.14	0.10
RH inferior frontal gyrus	13	40	26	10	^b^	0.17	0.10
RH precentral gyrus	44	47	4	11	^b^	0.11	0.09

*Notes*: in = in-group; out = out-group; Brodmann = Brodmann area; *x*, *y*, *z* = coordinates in MNI space; *k* = cluster size in mm^3^; max = maximum value within cluster; mean = average value within cluster; LH = left hemisphere; RH = right hemisphere. All analyses were *k*-threshold corrected at *P* < 0.001.

a Associated subclusters of LH dorsomedial PFC.

b Associated subclusters of RH anterior insula.

### Overall differences in functional activation for racial in-group *vs* out-group

**Fig. 4.**
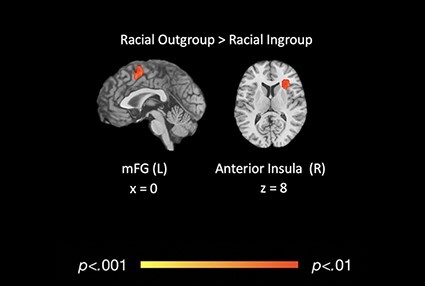
Regions of significant, consistent functional activation for overall racial in-group *vs* racial out-group social cognitive processing. These regions also reflect the meta-analytic contrasts of racial in-group *vs* racial out-group social cognitive processing: [(racial out-group*** ***>*** ***in-group)*** ***>*** ***(racial in-group*** ***>*** ***out-group)].

We next assessed the neural correlates of race-specific ingroup *vs* out-group social cognition. There were no clusters consistently activated across studies at the *P* < 0.001 threshold for ‘racial in-group > racial out-group’. However, ‘racial out-group > racial in-group’ (358/520 points; 80/116 contrasts) revealed two significant clusters of activity: one in left middle frontal gyrus (mFG; 0, 9, 44; *k* = 119), which extended into the mid-cingulate cortex (MCC), and one cluster in right anterior insula (40, 20, 13; *k* = 123), which extended into the claustrum and iFG (see Table 3 and Figure 4).

**Table 3.** Coordinates for overall differences in functional activation for racial in-group *vs* out-group

Region	Brodmann	*x*	*y*	*z*	*k*	Max	Mean
**Overall racial in-group > out-group**							
No significant clusters	n/a	n/a	n/a	n/a	n/a	n/a	n/a
**Overall racial out-group > in-group**							
*LH middle frontal gyrus (cluster)*	32	0	9	44	119	0.20	0.15
LH middle frontal gyrus	32	0	9	44	^a^	0.20	0.15
RH superior frontal gyrus	6	3	5	8	^a^	0.18	0.15
*RH anterior insula (cluster)*	45	40	20	13	123	0.20	0.15
RH anterior insula	45	40	20	13	^b^	0.20	0.15
RH anterior insula	13	39	20	5	^b^	0.18	0.15
RH claustrum		32	11	8	^b^	0.19	0.16

*Notes*: Brodmann = Brodmann area; *x*, *y*, *z* = coordinates in MNI space; *k* = cluster size in mm^3^; max = maximum value within cluster; mean = average value within cluster; LH = left hemisphere; R = right hemisphere. There were no significant in-group > out-group clusters. All analyses were *k*-threshold corrected at *P < *0.001.

a Associated subclusters of LH middle frontal gyrus.

b Associated subclusters of RH inferior frontal gyrus.

### Meta-analytic contrasts for racial in-group *vs* racial out-group

The meta-analytic contrasts for [(‘racial in-group > racial out-group’) > (‘racial out-group > racial in-group’)] and [(‘racial out-group >  racial in-group’) > (‘racial in-group > racial out-group’)] revealed a set of clusters similar to those identified in the primary neural reference space contrasts outlined above. There were no significant clusters of activation detected at *P* < 0.001 for [(‘racial in-group > racial out-group’) > (‘racial out-group > racial in-group’)] (358/520 points, 80/116 contrasts). The [(‘racial out-group > racial in-group’) > (‘racial in-group > racial out-group’)] (358/520 points, 80/116 contrasts) mirrored the same clusters of activation as the ‘racial out-group > racial in-group’ contrast: one in the left medial frontal gyrus (0, 9, 48; *k* = 119), and one in the right anterior insula, extending into the claustrum and iFG (36, 24, 9; *k* = 123; see Table 4).

**Table 4.** Coordinates for meta-analytic contrasts for overall racial in-group *vs* out-group

Region	Brodmann	*x*	*y*	*z*	*k*	Max	Mean
**Racial (in > out)) > racial (out > in)**							
No significant clusters	n/a	n/a	n/a	n/a	n/a	n/a	n/a
**Racial (out > in) > racial (in > out)**							
*LH medial frontal gyrus (cluster)*	32	0	9	44	119	0.20	0.15
LH medial frontal gyrus	32	0	9	44	^a^	0.20	0.15
RH superior frontal gyrus	6	3	5	58	^a^	0.18	0.15
*RH anterior insula (cluster)*	45	36	24	9	123	0.20	0.15
RH anterior insula	45	36	24	9	^b^	0.20	0.15
RH anterior insula	13	36	24	0	^b^	0.18	0.15
RH claustrum		30	15	3	^b^	0.19	0.16

*Notes*: in = in-group; out = out-group; Brodmann = Brodmann area; *x*, *y*, *z* = coordinates in MNI space; *k* = cluster size in mm^3^; max = maximum value within cluster; mean = average value within cluster; LH = left hemisphere; RH = right hemisphere. There were no significant in-group > out-group clusters. All analyses were *k*-threshold corrected at *P* < 0.001.

a Associated subclusters of LH middle frontal gyrus.

b Associated subclusters of RH anterior insula.

### Differences in functional activation for in-group *vs* out-group by social cognitive process

#### Empathy.

Next, we conducted analyses summarizing the neural reference spaces associated with ‘in-group empathy > out-group empathy’ and ‘out-group empathy > in-group empathy’ (64/520 points; 25/116 contrasts). The ‘in-group > out-group’ analysis revealed a large swathe of activation in the superior frontal gyrus with its peak in the left dmPFC (0, 49, 32; *k* = 100), bordering the left anterior medial PFC. The reverse contrast (i.e. ‘out-group > in-group empathy’; 64/520 points; 25/116 contrasts) showed three significant clusters of activation: one cluster in the left dorsolateral prefrontal cortex (dlPFC; −44, 38, 13; *k* = 256), a second cluster in the left premotor cortex (−27, 7, 50; *k* = 260) and a third cluster in the right precentral gyrus extending into the right supplementary motor area (SMA; 43, 22, 40; *k* = 250; see Table 5).

**Table 5.** Coordinates for differences in functional activation for in-group *vs* out-group by social cognitive process

Region	Brodmann	*x*	*y*	*z*	*k*	Max	Mean
**Empathy**							
**in-group > out-group**							
*LH dorsomedial PFC (cluster)*	9	0	49	32	100	0.11	0.09
LH dorsomedial PFC	9	0	49	32	^a^	0.11	0.09
LH dorsomedial PFC	10	0	60	28	^a^	0.10	0.09
**out-group > in-group**							
*LH dorsolateral PFC (cluster)*	46	−44	38	13	256	0.53	0.28
*LH premotor cortex (cluster)*	6	−27	7	50	260	0.52	0.52
**Emotion perception**							
**in-group > out-group**							
No significant clusters	n/a	n/a	n/a	n/a	n/a	n/a	n/a
**out-group > in-group**							
No significant clusters	n/a	n/a	n/a	n/a	n/a	n/a	n/a

*Notes*: Brodmann = Brodmann area; *x*, *y*, *z* = coordinates in MNI space; *k* = cluster size in mm^3^; max = maximum value within cluster; mean = average value within cluster; LH = left hemisphere; RH = right hemisphere. All analyses were *k*-threshold corrected at *P < *0.001.

a Associated subclusters of LH superior frontal gyrus.

#### Emotion perception.

There were no significant clusters of activation at *P* < 0.001 for ‘in-group > out-group emotion perception’ or ‘out-group > in-group emotion perception’ (140/520 points; 21/116 contrasts). These findings suggest that there were no core regions that consistently showed increased activity during in-group *vs* out-group (and vice versa) emotion perception across studies of social categories.

### Differences in functional activation for racial in-group *vs* out-group by social cognitive process

#### Empathy.

**Fig. 5.**
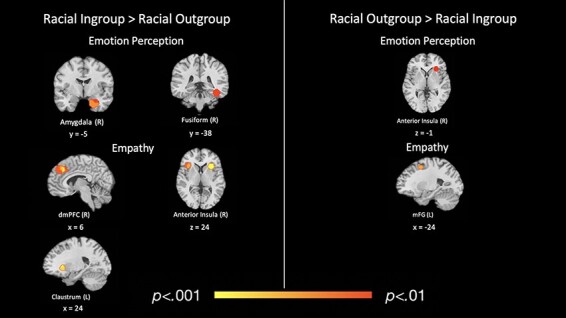
Regions of significant, consistent functional activation for racial in-group *vs* racial out-group empathy and emotion perception.

Finally, we conducted analyses summarizing the neural reference spaces associated with empathy and emotion perception specifically within our subset of racial in-group *vs* out-group contrasts. For ‘racial in-group empathy > racial out-group empathy’ (43/520 points; 20/116 contrasts), we found three significant clusters: one in the right dmPFC (7, 30, 34; *k* = 362), one in the right anterior insula (43, 20, 8; *k* = 260) and one in the claustrum (−23, 20, 6; *k* = 260). The reverse contrast (‘racial out-group empathy > racial in-group empathy’; 43/520 points; 20/116 contrasts) revealed one significant cluster located in the left mFG (−27, 7, 50; *k* = 260; see Table 6 and Figure 5).

**Table 6.** Coordinates for differences in functional activation for racial in-group *vs* out-group by social cognitive process

Region	Brodmann	*x*	*y*	*z*	*k*	Max	Mean
**Empathy**							
**Racial in-group > out-group**							
*RH dorsomedial PFC (cluster)*	9	7	30	34	362	0.40	0.20
RH dorsomedial PFC	9	7	30	34	^a^	0.40	0.20
RH dorsomedial PFC	8	15	34	42	^a^	0.22	0.18
*RH anterior insula (cluster)*	13	43	20	8	260	0.25	0.21
RH anterior insula	13	43	20	8	^b^	0.25	0.21
RH precentral gyrus	44	51	19	8	^b^	0.18	0.18
*LH claustrum (cluster)*		−23	20	6	260	0.35	0.31
**Racial out-group > in-group**							
*LH middle frontal gyrus (cluster)*	6	−27	7	50	260	0.52	0.52
**Emotion perception**							
**Racial in-group > out-group**							
*RH amygdala (cluster)*	20	37	−8	−19	269	0.34	0.30
*RH fusiform (cluster)*	37	50	−42	−8	239	0.23	0.23
**Racial out-group > in-group**							
*RH anterior insula (cluster)*	13	39	20	5	206	0.46	0.33
RH anterior insula	13	39	20	5	^c^	0.46	0.33
RH inferior frontal gyrus	13	40	26	12	^c^	0.33	0.33

*Notes*. Brodmann = Brodmann area; *x*, *y*, *z* = coordinates in MNI space; *k* = cluster size in mm^3^; max = maximum value within a cluster; mean = average value within cluster; LH = left hemisphere; RH = right hemisphere. All analyses were *k*-threshold corrected at *P* < 0.001.

a Associated subclusters of RH dorsomedial PFC.

b Associated subclusters of RH anterior insula.

c Associated subclusters of RH anterior insula.

#### Emotion perception.

For ‘racial in-group emotion perception > racial out-group emotion perception’ (112/520 points; 18/116 contrasts), there were significant clusters in the right amygdala (37, −8, −19; *k* = 269) and right fusiform gyrus (50, −42, −8; *k* = 239). For ‘racial out-group emotion perception > racial in-group emotion perception’ (112/520 points; 18/116 contrasts), there was one significant cluster in the right anterior insula (39, 20, 5; *k* = 206; see Table 6 and Figure 5). For written and tabular results for contrasts of other social cognitive processes, see Supplemental Materials Results and Supplementary Tables S3 and S4.

## Discussion

### Overall differences in functional activation for in-group *vs* out-group

In this meta-analysis, we examined the consistency and specificity of neural activation during intergroup social cognition. Results confirmed that there are consistent differences in neural activation during social cognition corresponding to whether the target of such cognition is an in-group *vs* out-group member. Across studies that engaged a variety of social cognitive processes and defined group membership based on diverse social categories, we found more consistent activation in prefrontal cortical regions including the right iFG, precentral gyrus and dmPFC when social cognition was directed at in-group (*vs* out-group) members. Moreover, meta-analytic contrasts indicated that the dmPFC was more consistently activated across study contrasts of ‘in-group > out-group’ compared to ‘out-group > in-group’ social cognition. Interestingly, the anterior insula was part of the neural reference space for social cognition regardless of whether the target of social cognition was an in-group or out-group member. However, subsequent meta-analytic contrasts revealed that although the anterior insula was consistently active across both ‘in-group > out-group’ and ‘out-group > in-group’ contrasts, this region was ‘more consistently’ activated for ‘out-group > in-group’ social cognitive processing relative to ‘in-group > out-group’ across studies.

Our overall in-group *vs* out-group findings offer some insight into neurocognitive processes that may underlie intergroup social behavior. For example, more consistent dmPFC activation during in-group (*vs* out-group) social cognition aligns with behavioral theories, suggesting that individuals are more likely to assign mental states to and act prosocially toward in-group compared to out-group members (Balliet *et al.*, 2014; Cikara *et al.*, 2014; Cikara and Van Bavel, 2014), as dmPFC activity has been consistently associated with the ability to infer mental states of others (Amodio and Frith, 2006; Frith and Frith, 2006; Saxe, 2006) and has been implicated in prosocial behavior (Telzer *et al.*, 2011; Waytz *et al.*, 2012). Further, research shows that the ability to simulate the minds of others tends to lead to greater prosocial behavior (Gaesser *et al.*, 2020). Notably, this consistent dmPFC activation for in-group relative to out-group members may seem surprising in light of existing literature suggesting a ventral–dorsal gradient in the mPFC, with the dmPFC implicated in social cognitive processing of dissimilar (e.g. out-group) others and the ventromedial PFC (vmPFC) implicated in processing of similar (e.g. in-group) others (Mitchell *et al.*, 2006; Lieberman *et al.*, 2019). However, other studies have offered evidence that such ventral–dorsal distinctions of the mPFC maybe be task-dependent (see Wagner *et al.*, 2012 for a review). Although the present analyses were not specifically intended to test the presence of a ventral–dorsal gradient in the mPFC, our results raise further questions about whether this gradient observed for some tasks generalizes across social cognition more broadly. As such, future work should explore this question more directly. Nevertheless, findings of the present study appear consistent with the notion that individuals are more likely to engage in mentalizing for in-group (*vs* out-group) members and that doing so may promote greater prosocial behavior (Balliet *et al.*, 2014; Telzer *et al.*, 2015), thus offering a potential neural mechanism underlying in-group favoritism.

Our finding of more consistent anterior insula activation during out-group social cognition also offers insight into the neural mechanisms that may underly out-group biases identified in the behavioral literature. Existing meta-analytic evidence suggests two major functional–anatomic subregions within the anterior insula: the ventral region, shown to be more active during visceral and affective experiences (especially subjective arousal), and the dorsal region, which is more associated with exogenous attention, including salience detection, attention orientation and task performance monitoring (Menon and Uddin, 2010; Touroutoglou *et al.*, 2012, 2016, 2018). Given these distinctions, one interpretation of the present findings is that out-group social cognition demands more attentional resources relative to in-group social cognition, perhaps because out-group members are more unfamiliar, infrequent or novel. This interpretation also corresponds with previous functional connectivity analyses that have shown evidence of anterior insula laterality during orientating/arousal and tasks requiring cognitive control. Specifically, right anterior insula has shown stronger connectivity with regions implicated in attentional orientation and arousal (e.g. postcentral gyrus and supramarginal gyrus), while left anterior insula shows stronger connectivity with regions implicated in perspective taking and cognitive motor control (e.g. dmPFC and superior frontal gyrus; Kann *et al.*, 2016). Along these lines, the right lateralization of anterior insula for out-group > in-group processing may reflect recruitment of attentional resources that results in focus on an individual’s salient out-group status, rather than individuating processes associated with greater medial prefrontal activation. On the other hand, the left lateralization of the anterior insula during in-group > out-group processing may facilitate communication with regions involved in perspective taking and mentalizing processes that allow for more individuated perceptions of in-group members. However, such interpretations are made cautiously given evidence, suggesting that functional lateralization of the anterior insula may vary with age, gender and other individual differences, and we were unable to account for these differences in the current analyses (Duerden *et al.*, 2013; Kann *et al.*, 2016).

Interestingly, the ‘out-group > in-group’ anterior insula findings also align with recent neuroimaging work showing that the right anterior insula is involved in integrating information about how others relate to one another in the service of making social group inferences (Lau *et al.*, 2020). For instance, in Lau *et al.* (2020), predictions about allyship among group members based on latent structure learning of social group coalitions were related to greater activation of the right anterior insula, compared to when predictions of allyship were based solely on similarity between targets. As such, the consistent anterior insula activation observed in our ‘out-group > in-group’ contrasts may suggest that, when considering out-group members, people engage in an additional layer of processing that incorporates how members of that out-group relate to other groups but do not engage in this same degree of processing when thinking about in-group members.

### Differences in functional activation for racial in-group *vs* racial out-group

We also investigated whether there are consistent neural differences in intergroup social cognition specifically within the social category of race. Interestingly, we did not find any regions consistently activated during racial in-group (*vs* out-group) social cognition. However, racial out-group (*vs* in-group) social cognitive processing was associated with more frequent activation of the mFG. We also found consistent anterior insula activation during racial out-group (*vs* in-group) social cognitive processing, mirroring the pattern of activation observed in the overall contrasts (i.e. ‘out-group > in-group’). A subsequent meta-analytic contrast comparing [(‘racial out-group > racial in-group’) > (‘racial in-group > racial out-group’)] revealed a significant cluster of activation in the right anterior insula that also closely mirrored the findings of the overall [(‘out-group in-group’) > (‘in-group > out-group’)] meta-analytic contrast, suggesting that the swathe of activation in this region may be associated with out-group processing in general, rather than being specific to racial out-group processing. Alternatively, this finding could be attributable to race being the most frequently investigated social category in the current literature, thus causing race-specific findings to drive an overall meta-analytic effect.

Our failure to identify consistent activation during racial in-group social cognition is interesting and suggests that there is heterogeneity in the brain areas underlying social cognition for racial in-group members across the literature. There were 80 contrasts in our database that addressed this particular question, so our failure to find consistent activation is not likely due to a lack of power. Indeed, a review of the individual contrast maps that contributed to these results revealed that the clusters of activation from individual studies were spatially heterogeneous, suggesting that the null results of these contrasts are driven by true variability in the data rather than due to lack of power. As such, one interpretation of these results is that social cognition for racial in-group members may be so routine that it does not preferentially activate brain regions above and beyond those activated for racial out-group members. In contrast, we did find that social cognitive processing directed at racial out-group individuals consistently elicits increased activity in regions implicated in exogenous attention and salience (e.g. anterior insula, mFG and iFG), mirroring findings for out-group members more generally and suggesting some consistency in regions involved in racial out-group social cognition across the literature.

### Differences in functional activation for overall in-group *vs* out-group and racial in-group *vs* out-group by social cognitive process

Finally, we explored how functional activation during intergroup processing may vary depending on the social cognitive process engaged, focusing specifically on empathy and emotion perception. We found empathy directed at in-group members was associated with more consistent activation in the dmPFC, even when focused specifically on racial in-group (*vs* out-group) empathy. Among racial in-group (*vs* out-group) empathy contrasts, we also observed a significant cluster of activation centered on the anterior insula. This cluster was more dorsal, which, in following with the ventral–dorsal distinctions of anterior insula functionality (Menon and Uddin, 2010; Touroutoglou *et al.*, 2012), suggests that empathy for in-group members may be more salient or elicit stronger attentional control (compared to out-group). However, these findings are difficult to interpret considering that our earlier results indicated that the anterior insula was more consistently activated in response to out-group members when we collapsed contrasts across all social cognition tasks. Still, this empathy-specific finding might suggest that in-group/out-group differences in activation of the dorsal anterior insula depend on the particular social cognitive process engaged. Conversely, empathy directed at out-group members was consistently associated with activity in motor (e.g. premotor cortex and precentral gyrus) and executive function areas (e.g. dlPFC and mFG) of the prefrontal cortex, perhaps suggesting that more effortful cognitive control is necessary to engage in empathy for out-group members.

We also observed differences in neural activation in response to racial in-group *vs* out-group members (although not to in-group *vs* out-group members in general) during emotion perception tasks. Specifically, perceiving emotions of racial in-group members was associated with activation in the amygdala and fusiform, regions that have been well-established in visual emotion perception (Pujol *et al.*, 2009; Dolcos *et al.*, 2011; Lindquist *et al.*, 2016), while emotion perception directed at racial out-group members was related to consistent anterior insula activation. Interestingly, this cluster was relatively more ventral than those observed in other out-group > in-group contrasts. One interpretation of this finding is that it may reflect greater aversive affective responding on the part of perceivers (Lindquist *et al.*, 2012, 2016), as perceivers may find emotional racial out-group members to be aversive. Again, these findings related to the activation of the anterior insula in these empathy and emotion perception contrasts remain difficult to interpret and warrant future studies to better understand how the ventral–dorsal anterior insula is operating in in-group *vs* out-group empathy and emotion perception. Nonetheless, these task-specific findings ultimately indicate that the neural correlates of intergroup social cognition do indeed vary depending on the specific social cognitive process engaged. This variation appears to be especially true for affective tasks like empathy and emotion perception, which would explain the inconsistencies noted in the literature regarding insula and dACC activity during in-group *vs* out-group social cognitive processing (Lee *et al.*, 2008; Xu *et al.*, 2009; Azevedo *et al.*, 2013; Cikara and Van Bavel, 2014; Liu *et al.*, 2015; Watson and de Gelder, 2017).

### Limitations and future directions

This work has some limitations. Data were constrained to published fMRI studies; therefore, it is unclear how the present results may be affected by publication bias. Moreover, the race-specific contrasts do not address how neural responding may vary depending on the specific racial groups involved (e.g. Black *vs* White and Asian *vs* Black). Distinguishing among various types of cross-race dyads is an important future direction, as different dynamics (e.g. cultural stereotypes and intergroup histories) exist for different racial/ethnic group pairings. Furthermore, due to a limited number of eligible studies, we were unable to assess in-group *vs* out-group differences in neural activation across all types of social cognition and social categories, thus leaving unanswered questions about other notable social cognitive processes such as theory of mind and perceptions of trustworthiness.

Finally, while our results may offer insight into neural mechanisms underlying intergroup social behaviors, they are subject to important caveats inherent in any neuroimaging meta-analysis. First, coordinate-based neuroimaging meta-analyses are the gold standard when aggregating across the neuroimaging literature; yet, these techniques rely on functional coordinates derived from contrast analyses but do not incorporate coordinates derived from correlational or functional connectivity analyses, thus limiting the kinds of studies that can be included in the database. Nonetheless, this meta-analysis helps reveal which regions are most consistently active for different social cognitive processes and targets, which may in turn prove useful for future studies using more advanced techniques such as functional connectivity. Second, interpretation of meta-analytic neuroimaging data is subject to reverse inference—inferring cognitive processes from the presence of neural activation (Poldrack, 2011). Future studies should follow up on these interpretations using experimental designs that pinpoint brain–behavior links. Finally, our findings do not provide evidence of a causal link between neural activation and subsequent behavior in intergroup contexts. Future research might explore how inducing neural activity in the regions identified here may impact individuals’ behaviors when directed at in-group *vs* out-group members.

## Conclusion

We conducted the largest meta-analysis to date of the fMRI literature examining the neural correlates of social cognition across group lines. Our findings align with existing behavioral data and theories on intergroup social phenomena (e.g. in-group favoritism and out-group degradation) and help clarify how the brain gives rise to diverse social cognitive processes, which in turn may manifest as biased social behaviors in intergroup contexts. We hope this work can help guide future research and interventions that address intergroup behavioral dynamics.

## Supplementary Material

nsab034_Supp

## References

[R1] Adams R.B., Rule N.O., Franklin R.G., et al. (2010). Cross-cultural reading the mind in the eyes: an fMRI investigation. *Journal of Cognitive Neuroscience*, 22(1), 97–108. 19199419 10.1162/jocn.2009.21187

[R2] Amodio D.M., Frith C.D. (2006). Meeting of minds: the medial frontal cortex and social cognition. *Nature Reviews Neuroscience*, 7(4), 268–77. 16552413 10.1038/nrn1884

[R3] Azevedo R.T., Macaluso E., Avenanti A., Santangelo V., Cazzato V., Aglioti S.M. (2013). Their pain is not our pain: brain and autonomic correlates of empathic resonance with the pain of same and different race individuals. *Human Brain Mapping*, 34(12), 3168–81. 22807311 10.1002/hbm.22133 PMC6870096

[R4] Balliet D., Wu J., De Dreu C.K.W. (2014). in-group favoritism in cooperation: a meta-analysis. *Psychological Bulletin*, 140(6), 1556–81. 25222635 10.1037/a0037737

[R5] Brauer M. (2001). Intergroup perception in the social context: the effects of social status and group membership on perceived out-group homogeneity and ethnocentrism. *Journal of Experimental Social Psychology*, 37(1), 15–31.

[R6] Brewer M.B. (1999). The psychology of prejudice: in-group love and out-group hate? *Journal of Social Issues*, 55(3), 429–44.

[R7] Brewer M.B. (2007). *The social psychology of intergroup relations: social categorization, in-group bias, and out-group prejudice. In Social psychology: handbook of basic principles*.pp. 695–715.

[R8] Cikara M., Bruneau E., Van Bavel J.J., Saxe R. (2014). Their pain gives us pleasure: how intergroup dynamics shape empathic failures and counter-empathic responses. *Journal of Experimental Social Psychology*, 55, 110–25. 25082998 10.1016/j.jesp.2014.06.007 PMC4112600

[R9] Cikara M., Van Bavel J.J., Ingbretsen Z.A., Lau T. (2017). Decoding “us” and “them”: neural representations of generalized group concepts. *Journal of Experimental Psychology. General*, 146(5), 621–31. 28459261 10.1037/xge0000287

[R10] Cikara M., Van Bavel J.J. (2014). The neuroscience of intergroup relations: an integrative review. *Perspectives on Psychological Science: A Journal of the Association for Psychological Science*, 9(3), 245–74. 26173262 10.1177/1745691614527464

[R0010a] Cremers H.R., Wager T.D., Yarkoni T. (2017). The relation between statistical power and inference in fMRI. *Plos One*, 12(11), p. e0184923. 10.1371/journal.pone.0184923 PMC5695788 29155843

[R11] Cunningham W.A., Johnson M.K., Raye C.L., Chris Gatenby J., Gore J.C., Banaji M.R. (2004). Separable neural components in the processing of black and white faces. *Psychological Science*, 15(12), 806–13. 15563325 10.1111/j.0956-7976.2004.00760.x

[R12] Dolcos F., Iordan A.D., Dolcos S. (2011). Neural correlates of emotion-cognition interactions: a review of evidence from brain imaging investigations. *Journal of Cognitive Psychology (Hove, England)*, 23(6), 669–94. 10.1080/20445911.2011.594433 PMC3206704 22059115

[R13] Drwecki B.B., Moore C.F., Ward S.E., Prkachin K.M. (2011). Reducing racial disparities in pain treatment: the role of empathy and perspective-taking. *Pain*, 152(5), 1001–6. 21277087 10.1016/j.pain.2010.12.005

[R14] Duerden E.G. Arsalidou M., Lee M., Taylor M.J. (2013). Lateralization of affective processing in the insula. *Neuroimage*, 78, 159–75. 23587690 10.1016/j.neuroimage.2013.04.014

[R15] Frith C.D., Frith U. (2006). The neural basis of mentalizing. *Neuron*, 50(4), 531–4. 16701204 10.1016/j.neuron.2006.05.001

[R16] Gaesser B., Shimura Y., Cikara M. (2020). Episodic simulation reduces intergroup bias in prosocial intentions and behavior. *Journal of Personality and Social Psychology*, 118(4), 683–705. 31157527 10.1037/pspi0000194

[R17] Han S. (2018). Neurocognitive basis of racial in-group bias in empathy. *Trends in Cognitive Sciences*, 22(5), 400–21. 29563059 10.1016/j.tics.2018.02.013

[R18] Harris L.T., Fiske S.T. (2006). Dehumanizing the lowest of the low: neuroimaging responses to extreme out-groups. *Psychological Science*, 17(10), 847–53. 17100784 10.1111/j.1467-9280.2006.01793.x

[R19] Hart A.J., Whalen P.J., Shin L.M., McInerney S.C., Fischer H., Rauch S.L. (2000). Differential response in the human amygdala to racial out-group vs in-group face stimuli. *Neuroreport*, 11(11), 2351–5. 10943684 10.1097/00001756-200008030-00004

[R20] Hein G., Silani G., Preuschoff K., Batson C.D., Singer T. (2010). Neural responses to in-group and out-group members’ suffering predict individual differences in costly helping. *Neuron*, 68(1), 149–60. 20920798 10.1016/j.neuron.2010.09.003

[R21] Hughes B.L., Camp N.P., Gomez J., Natu V.S., Grill-Spector K., Eberhardt J.L. (2019). Neural adaptation to faces reveals racial out-group homogeneity effects in early perception. *Proceedings of the National Academy of Sciences of the United States of America*, 116(29), 14532–7. 31262811 10.1073/pnas.1822084116 PMC6642392

[R22] Johnson J.D., Simmons C.H., Jordav A., et al. (2002). Rodney King and O. J. revisited: the impact of race and defendant empathy induction on judicial decisions. *Journal of Applied Social Psychology*, 32(6), 1208–23.

[R23] Judd C.M., Park B. (1988). Out-group homogeneity: judgments of variability at the individual and group levels. *Journal of Personality and Social Psychology*, 54(5), 778–88.

[R24] Kann S., Zhang S., Manza P., Leung H.-C., Li C.-S.R. (2016). Hemispheric lateralization of resting-state functional connectivity of the anterior insula: association with age, gender, and a novelty-seeking trait. *Brain Connectivity*, 6(9), 724–34. 27604154 10.1089/brain.2016.0443 PMC5105339

[R25] Kaseweter K.A., Drwecki B.B., Prkachin K.M. (2012). Racial differences in pain treatment and empathy in a Canadian sample. *Pain Research & Management*, 17(6), 381–4. 23248809 10.1155/2012/803474 PMC3659010

[R26] Kober H., Wager T.D. (2010). Meta-analysis of neuroimaging data. *Wiley Interdisciplinary Reviews: Cognitive Science*, 1(2), 293–300. 24052810 10.1002/wcs.41 PMC3775366

[R27] Lau T., Gershman S.J., Cikara M. (2020). Social structure learning in human anterior insula. *eLife*, 9, e53162. 10.7554/eLife.53162 PMC7136019 32067635

[R28] Lau T., Cikara M. (2017). fMRI repetition suppression during generalized social categorization. *Scientific Reports*, 7(1), 4262. 10.1038/s41598-017-04115-8 PMC5487342 28655903

[R29] Lee K.-U., Khang H.S., Kim K.-T., et al. (2008). Distinct processing of facial emotion of own-race versus other-race. *Neuroreport*, 19(10), 1021–5. 18580572 10.1097/WNR.0b013e3283052df2

[R30] Liberati A., Altman D.G., Tetzlaff J., et al. (2009). The PRISMA statement for reporting systematic reviews and meta-analyses of studies that evaluate health care interventions: explanation and elaboration. *Journal of Clinical Epidemiology*, 62(10), e1–34. 19631507 10.1016/j.jclinepi.2009.06.006

[R31] Liberman Z., Woodward A.L., Kinzler K.D. (2017). The origins of social categorization. *Trends in Cognitive Sciences*, 21(7), 556–68. 28499741 10.1016/j.tics.2017.04.004 PMC5605918

[R32] Lieberman M.D., Straccia M.A., Meyer M.L., Du M., Tan K.M. (2019). Social, self, (situational), and affective processes in medial prefrontal cortex (MPFC): causal, multivariate, and reverse inference evidence. *Neuroscience and Biobehavioral Reviews*, 99, 311–28. 30610911 10.1016/j.neubiorev.2018.12.021

[R33] Lindquist K.A., Wager T.D., Kober H., Bliss-Moreau E., Barrett L.F. (2012). The brain basis of emotion: a meta-analytic review. *Behavioral and Brain Sciences*, 35(3), 121–43. 10.1017/S0140525X11000446 PMC4329228 22617651

[R34] Lindquist K.A., Satpute A.B., Wager T.D., Weber J., Barrett L.F. (2016). The brain basis of positive and negative affect: evidence from a meta-analysis of the human neuroimaging literature. *Cerebral Cortex*, 26(5), 1910–22. 25631056 10.1093/cercor/bhv001 PMC4830281

[R35] Liu Y., Lin W., Xu P., Zhang D., Luo Y. (2015). Neural basis of disgust perception in racial prejudice. *Human Brain Mapping*, 36(12), 5275–86. 26417673 10.1002/hbm.23010 PMC6868979

[R36] MacCormack J., Stein A., Kang J., Giovanello K., Satpute A., Lindquist K. (2020). Affect in the aging brain: a neuroimaging meta-analysis of older vs. younger. *Affective Science*, 1(3), 128–54. 10.1007/s42761-020-00016-8 PMC9382982 36043210

[R37] Mahajan N., Wynn K. (2012). Origins of “us” versus “them”: prelinguistic infants prefer similar others. *Cognition*, 124(2), 227–33. 22668879 10.1016/j.cognition.2012.05.003

[R38] Major B., Mendes W.B., Dovidio J.F. (2013). Intergroup relations and health disparities: a social psychological perspective. *Health Psychology*, 32(5), 514–24. 23646834 10.1037/a0030358 PMC3988903

[R39] Mathur V.A., Harada T., Lipke T., Chiao J.Y. (2010). Neural basis of extraordinary empathy and altruistic motivation. *Neuroimage*, 51(4), 1468–75. 20302945 10.1016/j.neuroimage.2010.03.025

[R40] Menon V., Uddin L.Q. (2010). Saliency, switching, attention and control: a network model of insula function. *Brain Structure & Function*, 214(5–6), 655–67. 20512370 10.1007/s00429-010-0262-0 PMC2899886

[R41] Mitchell J.P., Macrae C.N., Banaji M.R. (2006). Dissociable medial prefrontal contributions to judgments of similar and dissimilar others. *Neuron*, 50(4), 655–63. 16701214 10.1016/j.neuron.2006.03.040

[R42] Molenberghs P., Louis W.R. (2018). Insights from fMRI studies into in-group bias. *Frontiers in Psychology*, 9, 1868. 10.3389/fpsyg.2018.01868 PMC6174241 30327636

[R43] Ostrom T.M., Sedikides C. (1992). Out-group homogeneity effects in natural and minimal groups. *Psychological Bulletin*, 112(3), 536–52.

[R44] Phelps E.A., O’Connor K.J., Cunningham W.A., et al. (2000). Performance on indirect measures of race evaluation predicts amygdala activation. *Journal of Cognitive Neuroscience*, 12(5), 729–38. 11054916 10.1162/089892900562552

[R45] Poldrack R.A. (2011). Inferring mental states from neuroimaging data: from reverse inference to large-scale decoding. *Neuron*, 72(5), 692–7. 22153367 10.1016/j.neuron.2011.11.001 PMC3240863

[R46] Pujol J., Harrison B.J., Ortiz H., et al. (2009). Influence of the fusiform gyrus on amygdala response to emotional faces in the non-clinical range of social anxiety. *Psychological Medicine*, 39(7), 1177–87. 19154647 10.1017/S003329170800500X

[R47] Richeson J. Dovidio J.F. Shelton J.N. Hebl M. (2007). Implications of in-group-out-group membership for interpersonal perceptions: faces and emotion. In: Hess, U., Philippot, P., editors. *Group Dynamics and Emotional Expression*, Cambridge: Cambridge University Press, 7–32.

[R48] Saxe R. (2006). Uniquely human social cognition. *Current Opinion in Neurobiology*, 16(2), 235–9. 16546372 10.1016/j.conb.2006.03.001

[R49] Shkurko A.V. (2013). Is social categorization based on relational in-group/out-group opposition? A meta-analysis. *Social Cognitive and Affective Neuroscience*, 8(8), 870–7. 22847948 10.1093/scan/nss085 PMC3831554

[R50] Tajfel H. (1982). Social psychology of intergroup relations. *Annual Review of Psychology*, 33(1), 1–39.

[R51] Tajfel H., Turner J. (1979). An integrative thoery of intergroup conflict. *Organizational Identity*, 56(65), 33–47.

[R52] Telzer E.H., Masten C.L., Berkman E.T., Lieberman M.D., Fuligni A.J. (2011). Neural regions associated with self control and mentalizing are recruited during prosocial behaviors towards the family. *Neuroimage*, 58(1), 242–9. 21703352 10.1016/j.neuroimage.2011.06.013 PMC3276247

[R53] Telzer E.H., Ichien N., Qu Y. (2015). The ties that bind: group membership shapes the neural correlates of in-group favoritism. *Neuroimage*, 115, 42–51. 25913702 10.1016/j.neuroimage.2015.04.035

[R54] Touroutoglou A., Hollenbeck M., Dickerson B.C., Feldman Barrett L. (2012). Dissociable large-scale networks anchored in the right anterior insula subserve affective experience and attention. *Neuroimage*, 60(4), 1947–58. 22361166 10.1016/j.neuroimage.2012.02.012 PMC3345941

[R55] Touroutoglou A., Bliss-Moreau E., Zhang J., et al. (2016). A ventral salience network in the macaque brain. *Neuroimage*, 132, 190–7. 26899785 10.1016/j.neuroimage.2016.02.029 PMC4851897

[R56] Touroutoglou A., Zhang J., Andreano J.M., Dickerson B.C., Barrett L.F. (2018). Dissociable effects of aging on salience subnetwork connectivity mediate age-related changes in executive function and affect. *Frontiers in Aging Neuroscience*, 10, 410. 10.3389/fnagi.2018.00410 PMC6304391 30618717

[R0057a] Turner B.O., Paul E.J., Miller M.B and Barbey A.K, (2018). Small sample sizes reduce the replicability of task-based fMRI studies. *Communications Biology*, 1 p. 62. 10.1038/s42003-018-0073-z PMC6123695 30271944

[R57] Van Bavel J.J., Packer D.J., Cunningham W.A. (2008). The neural substrates of in-group bias: a functional magnetic resonance imaging investigation. *Psychological Science*, 19(11), 1131–9. 19076485 10.1111/j.1467-9280.2008.02214.x

[R58] Van Hoorn J., Shablack H., Lindquist K.A., Telzer E.H. (2019). Incorporating the social context into neurocognitive models of adolescent decision-making: a neuroimaging meta-analysis. *Neuroscience and Biobehavioral Reviews*, 101, 129–42. 31006540 10.1016/j.neubiorev.2018.12.024 PMC6659412

[R59] Wager T.D., Lindquist M., Kaplan L. (2007). Meta-analysis of functional neuroimaging data: current and future directions. *Social Cognitive and Affective Neuroscience*, 2(2), 150–8. 18985131 10.1093/scan/nsm015 PMC2555451

[R60] Wagner D.D., Haxby J.V., Heatherton T.F. (2012). The representation of self and person knowledge in the medial prefrontal cortex. *Wiley Interdisciplinary Reviews: Cognitive Science*, 3(4), 451–70. 22712038 10.1002/wcs.1183 PMC3375705

[R61] Watson R., de Gelder B. (2017). How white and black bodies are perceived depends on what emotion is expressed. *Scientific Reports*, 7, 41349. 10.1038/srep41349 PMC5269713 28128279

[R62] Waytz A., Zaki J., Mitchell J.P. (2012). Response of dorsomedial prefrontal cortex predicts altruistic behavior. *The Journal of Neuroscience*, 32(22), 7646–50. 22649243 10.1523/JNEUROSCI.6193-11.2012 PMC3387686

[R63] Xu X., Zuo X., Wang X., Han S. (2009). Do you feel my pain? Racial group membership modulates empathic neural responses. *The Journal of Neuroscience*, 29(26), 8525–9. 19571143 10.1523/JNEUROSCI.2418-09.2009 PMC6665679

[R64] Zaki J., Cikara M. (2015). Addressing empathic failures. *Current Directions in Psychological Science*, 24(6), 471–6.

