# Supplementary material for: The neural underpinnings of intergroup social cognition: an fMRI meta-analysis
Source: Soc Cogn Affect Neurosci. 2021 Mar 24;16(9):903–14. doi: 10.1093/scan/nsab034 (PMC8421705; doi:10.1093/scan/nsab034)
Supplement: nsab034_Supp [file nsab034_supp.zip › suppl.docx]

**Supplementary Materials**

**Methods**

**Search Terms**

Search terms for identifying articles: “fMRI + ingroup + outgroup + political (politics)”; “fMRI + ingroup + outgroup + religion”; “fMRI + ingroup + outgroup + gender”; “fMRI + ingroup + outgroup + social status”; “fMRI + ingroup + outgroup + race”. Hyphenated versions of ingroup (i.e., in-group) and outgroup (i.e., out-group) were included in searches. We also ran searches excluding the “ingroup + outgroup”/ “group membership” terms and using only “fMRI + [social category]”, “fMRI + [social category], + bias” or fMRI + [social category] + affiliation”. This ensured that we captured all possible studies examining different social categories.

**Data Analysis: Overview and Rationale**

To address the aims of this study, we used Multilevel Kernel Density Analysis (Kober et al., 2008; Kober & Wager, 2010; Wager, Lindquist, & Kaplan, 2007), which is a coordinate-based fMRI meta-analysis technique that computes meta-analytic summary contrasts of brain regions that are more reliably active above beyond what would be expected by chance during one condition versus another (e.g. *ingroup > outgroup*) across the included studies. The MKDA procedure nests reported peak coordinates within contrast maps, thus making the contrast maps the unit of analysis and treating them as random effects. This method is advantageous over other methods in which the peak coordinates are the unit of analysis because it prevents a single study from biasing the results if the study reports several nearby peaks (Kober et al., 2008). Additionally, the MKDA method accounts for both study quality and sample by weighting studies based on their sample size and their use of fixed vs. random effects analysis. Specifically, MKDA down-weights study contrasts models that used fixed effects modeling in their analyses, as fixed effects are unable to generalize to the population. By weighting studies in this manner, MKDA allows for higher-quality (i.e., higher powered, more generalizable) studies to have greater impact on the meta-analytic results, as random effects analysis allows (Kober & Wagner, 2010).

A general rule of thumb is that a MKDA with <10 contrasts is unreliable (van Hoorn et al., 2019; Lindquist et al., 2016), so we only ran supplemental analyses for individual social cognitive processes when there were more than 10 contrasts. Processes that met this criterion were: Social Perception, Social Categorization, and Impression Formation (see **SM Table 1** for how these processes/task types were defined).

**Results**

**Differences in functional activation for ingroup vs. outgroup by social cognitive process**

We conducted supplemental analyses examining differences in functional activation during additional social cognitive processes identified in the literature, including social perception, social categorization, and impression formation tasks (see **SM Table 3**). Of note, findings reported here should be interpreted with caution given the limited number of contrasts contributing to the results.

**Social perception tasks.** Among studies in which participants passively viewed ingroup and outgroup members or completed an unrelated task not involving categorization according to group membership (e.g., “viewing Black/White faces and indicating on which side of the screen the face appeared”), the *ingroup > outgroup* comparison (122/520 points; 22/116 contrasts) revealed one cluster of activation with its peak in the right inferior occipitotemporal gyrus (53, -68, 2; *k*=199, *p<*.001) that extended into the right fusiform gyrus. The contrast of *outgroup > ingroup social perception* (122/520 points; 22/116 contrasts) showed one cluster of activation centered in the right dorsal anterior cingulate cortex (dACC; 3, 21, 31; *k*=146, *p<*.001). No other significant clusters were observed.

**Social categorization tasks**. Among studies that explicitly instructed participants to categorize stimuli based on their group membership, an analysis of *ingroup > outgroup* (29/520 points; 12/116 contrasts) revealed one cluster of activation with its peak in the right iFG (50, 6, 33; *k*=179, *p<*.001). There were no significant regions of activation at *p<.*001 for *outgroup > ingroup social categorization.*

**Impression formation tasks.** Among studies in which participants were explicitly instructed to generate impressions of ingroup and outgroup others, there were no significant cluster of activation at *p* < .001 for *ingroup > outgroup impression formation*. However, the *outgroup > ingroup impression formation* comparison (28/520 points; 11/116 contrasts) revealed three significant clusters of activation: one in the right anterior medial prefrontal cortex (amPFC; 22, 57, 7; *k*= 256, *p* <.001), one in the right middle temporal gyrus (45, -55,11; *k*= 257, *p* <.001), and one in the right middle occipital gyrus extending into the fusiform gyrus (48, -98, 0; *k*=257, *p* <.001).

**Differences in functional activation for racial ingroup vs. racial outgroup by social cognitive process**

For our race-specific contrasts, there were no significant *racial ingroup > outgroup* or *racial outgroup > ingroup* clusters for social categorization and impression formation tasks. However, we did find significant differences in activation for social perception tasks. The contrast of *racial ingroup > outgroup social perception* (77/520 points, 20/116 contrasts) revealed two significant clusters: one in the right dorsal anterior cingulate cortex (dACC; 7, 37,-11; *k*=266, *p* <.001) and one in the cerebellum (-7, -79, -25; *k*=262, *p* <.001). The *racial outgroup > ingroup social perception* contrast (77/520 points, 20/116 contrasts) again revealed significant activation in the right dACC, which extended into the mid-cingulate cortex (3, 21, 31; *k=*146, *p* <.001). (see **SM Table 4**)

**SM Table 1. List and Description of Task Types**

| **Construct Name**  **Empathy**  **Emotion Perception**  **Social Perception**  **Social Categorization**  **Impression Formation**  Individuation  Imitation  Imitation Regulation  Theory of Mind  Prosociality  Resource Allocation  Delivering Reward  Perception of Harm  Social Exclusion  Trust  Memory Recall | **Description of Tasks**  Participants view ingroup/outgroup members in pain  Participants view emotional ingroup/outgroup stimuli  Participants passively view ingroup/outgroup members or complete an unrelated task while viewing ingroup/outgroup members  Participants explicitly categorize stimuli according to ingroup/outgroup membership  Participants generating impressions of ingroup/outgroup members  Participants receive individuating information about ingroup/outgroup member or engage with stimuli in a way that leads to individuation of ingroup/outgroup other  Participants imitate ingroup/outgroup members’ gestures  Participants suppress (as opposed to follow) tendency to imitate ingroup/outgroup members’ gestures  Participants perform mentalizing or perspective-taking tasks for ingroup/outgroup members  Participants decide whether to act prosocially toward ingroup/outgroup other  Participants make decisions about allocating resources among a group of ingroup/outgroup members  Participants deliver reward to ingroup/outgroup member  Participants perceive ingroup/outgroup members perpetrating harm against others  Participants are socially excluded by ingroup/outgroup members  Participants play a trust game with ingroup/outgroup members  Participants subsequent memory recall (versus forgetting) of ingroup/outgroup member stimuli | **Example Task**  Viewing individuals of same/other race being touched by needle  Viewing faces of same/ other race individuals with angry/happy/neutral expressions  Viewing images of individuals in same/opposing political parties  Viewing faces of same/other team members and categorizing faces according to team membership  Forming a quick impression of same/other race individuals based on face images  Judging same/other race individuals on “friendliness” based on an image and brief descriptive of the individual  Imitating same/other gender actors performing hand gestures and  Refraining from imitating same/other race actors’ facial expressions and hand gestures  Reading the Mind in the Eyes Task with same/other race eyes  Modified Dictator Game with same/other culture confederate  Distributing money to same/other team members  Administering a monetary reward or electric shock punishment to a student from same/other university  Viewing images of student from same/other university harming another student from same/other university  Cyberball with same/other race confederates  Deciding whether to “trust” an individual from same/other political party to carry out risky investment or keep investment to self  Viewing images of same/other race faces and recalling those faces 24 hours later | **Number of Contrasts**  25  22  12  21  12  2  7  2  3  3  1  1  1  1  1  1 |
| --- | --- | --- | --- |

**Notes.** Only bolded task types were included in analyses as other tasks had too few contrasts to provide reliable meta-analytic estimates.

**SM Table 2. List of references included in the meta-analysis and their corresponding social category and social cognition process engaged**

| **Author** | **Social Category** | **Task Type** |
| --- | --- | --- |
| Adams et al. (2010) | Race | Theory of Mind |
| Azevedo et al. (2013) | Race | Social Perception, Empathy |
| Berlingeri et al. (2016) | Race | Empathy |
| Bestelmeyer et al. (2015) | Culture | Social Categorization |
| Brown et al. (2017) | Race | Memory Recall |
| Cao et al. (2015) | Race | Empathy |
| Cheon et al. (2013) | Culture | Empathy |
| Chiao et al. (2008) | Culture | Emotion Perception |
| Contreras et al. (2013) | Race | Empathy |
| Cunningham et al. (2004) | Race | Social Perception |
| Earls et al. (2013) | Race | Imitation, Social Perception |
| Falk et al. (2012) | Political | Theory of Mind |
| Feng et al. (2011) | Race | Social Categorization |
| Freeman et al. (2010) | Race | Individuation |
| Hart et al. (2000) | Race | Social Perception |
| Hein et al. (2010) | Minimal | Empathy |
| Junger et al. (2013) | Gender | Social Categorization |
| Kaplan et al. (2007) | Political | Social Perception |
| Krautheim et al. (2018) | Minimal | Emotion Perception |
| Krill et al. (2009) | Race | Social Exclusion |
| Lee et al. (2008) | Race | Social Perception |
| Li et al. (2015) | Race | Emotion Perception |
| Li et al. (2016) | Race | Impression Formation |
| Liu et al. (2015) | Race | Emotion Perception |
| Losin et al. (2014) | Race | Imitation |
| Losin et al. (2012) | Race | Imitation |
| Losin et al. (2012) | Gender | Imitation |
| Luo et al. (2015) | Race | Empathy |
| Mathur et al. (2010) | Race | Social Perception |
| Mattan et al. (2018) | Race | Impression Formation |
| Molenberghs et al. (2017) | Political | Impression Formation |
| Molenberghs et al. (2016) | Minimal | Perception of Harm |
| Molenberghs et al. (2014) | Minimal | Delivering Reward |
| Molenberghs et al. (2014) | Minimal | Social Categorization |
| Morrison et al. (2012) | Minimal | Social Categorization |
| Rauchbauer et al. (2015) | Race | Imitation Regulation |
| Richeson et al. (2008) | Race | Social Perception |
| Richeson et al. (2003) | Race | Social Perception |
| Richins et al. (2019) | Minimal | Social Perception, Empathy |
| Ronquillo et al. (2007) | Race | Social Perception |
| Ruckmann et al. (2015) | Minimal | Empathy |
| Rule et al. (2010) | Culture, Political | Impression Formation |
| Sheng et al. (2014) | Race | Emotion Perception |
| Telzer et al. (2015) | Race | Prosociality |
| Van Bavel et al. (2008) | Race, Minimal | Social Categorization |
| Volz et al. (2009) | Minimal | Resource Allocation |
| Watson et al. (2017) | Race | Emotion Perception |
| Wheeler et al. (2005) | Race | Individuation |
| Wu et al. (2018) | Political | Trust |
| Xu et al. (2009) | Race | Empathy |

**SM Table 3. Coordinates for differences in functional activation for ingroup vs. outgroup by social cognitive process**

| ***Region*** | ***Brodmann*** | ***x*** | ***y*** | ***z*** | ***k*** | ***max*** | ***mean*** |
| --- | --- | --- | --- | --- | --- | --- | --- |
| **Social Perception** |  |  |  |  |  |  |  |
| ***Ingroup > Outgroup*** |  |  |  |  |  |  |  |
| **RH Occipitotemporal Gyrus (cluster)** | n/a | **53** | **-68** | **2** | **199** | **.52** | **.43** |
|  |  |  |  |  |  |  |  |
| ***Outgroup > Ingroup*** |  |  |  |  |  |  |  |
| **RH Dorsal ACC (cluster)** | **32** | **3** | **21** | **31** | **146** | **.44** | **.31** |
| RH Dorsal ACC | 32 | 3 | 21 | 31 | ^a^ | .44 | .32 |
| RH Dorsal ACC | 32 | 14 | 20 | 37 | ^a^ | .23 | .23 |
|  |  |  |  |  |  |  |  |
| **Social Categorization** |  |  |  |  |  |  |  |
| ***Ingroup > Outgroup*** |  |  |  |  |  |  |  |
| **RH Inferior Frontal Gyrus (cluster)** | **9** | **50** | **6** | **33** | **179** | **.27** | **.26** |
|  |  |  |  |  |  |  |  |
| ***Outgroup > Ingroup*** |  |  |  |  |  |  |  |
| No Significant Clusters | n/a | n/a | n/a | n/a | n/a | n/a | n/a |
|  |  |  |  |  |  |  |  |
| **Impression Formation** |  |  |  |  |  |  |  |
| ***Ingroup > Outgroup*** |  |  |  |  |  |  |  |
| No Significant Clusters | n/a | n/a | n/a | n/a | n/a | n/a | n/a |
|  |  |  |  |  |  |  |  |
| ***Outgroup > Ingroup*** |  |  |  |  |  |  |  |
| **RH Anterior Medial PFC (cluster)** | **10** | **22** | **57** | **7** | **256** | **.22** | **.22** |
| **RH Middle Temporal Gyrus (cluster)** | **39** | **45** | **-55** | **11** | **257** | **.22** | **.22** |
| **RH Middle Occipital Gyrus (cluster)** | **19** | **48** | **-98** | **0** | **257** | **.22** | **.22** |

**Notes**. *Brodmann* = Brodmann area; *x, y, z* = coordinates in Montreal Neurological Institute (MNI) space; *k* = cluster size in mm^3^; *max* = maximum value within cluster; *mean* = average value within cluster. L = left, R = right. ^a^ = associated subclusters of RH Dorsal ACC. There were no significant Ingroup > Outgroup or Outgroup > Ingroup clusters for Emotion Perception. There were no significant Outgroup > Ingroup clusters for Social Categorization. There were no significant Ingroup > Outgroup clusters for Impression Formation tasks. All analyses were k-threshold corrected at *p<*.001.

**SM Table 4. Coordinates for differences in functional activation for racial ingroup vs. outgroup by social cognitive process**

| ***Region*** | ***Brodmann*** | ***x*** | ***y*** | ***z*** | ***k*** | ***max*** | ***mean*** |
| --- | --- | --- | --- | --- | --- | --- | --- |
|  |  |  |  |  |  |  |  |
| **Social Perception** |  |  |  |  |  |  |  |
| ***Racial Ingroup > Outgroup*** |  |  |  |  |  |  |  |
| **RH Dorsal ACC (cluster)** | **32** | **7** | **37** | **-11** | **266** | **.20** | **.20** |
| **Cerebellum (cluster)** |  | **-7** | **-79** | **-25** | **262** | **.20** | **.20** |
|  |  |  |  |  |  |  |  |
| ***Racial Outgroup > Ingroup*** |  |  |  |  |  |  |  |
| **RH Dorsal ACC (cluster)** | **32** | **3** | **21** | **31** | **146** | **.44** | **.31** |
| RH Dorsal ACC | 32 | 3 | 21 | 31 | ^a^ | .44 | .32 |
| RH Dorsal ACC | 32 | 14 | 20 | 37 | ^a^ | .23 | .23 |
|  |  |  |  |  |  |  |  |
| **Social Categorization** |  |  |  |  |  |  |  |
| ***Racial Ingroup > Outgroup*** |  |  |  |  |  |  |  |
| No Significant Clusters | n/a | n/a | n/a | n/a | n/a | n/a | n/a |
|  |  |  |  |  |  |  |  |
| ***Racial Outgroup > Ingroup*** |  |  |  |  |  |  |  |
| No Significant Clusters | n/a | n/a | n/a | n/a | n/a | n/a | n/a |
|  |  |  |  |  |  |  |  |
| **Impression Formation** |  |  |  |  |  |  |  |
| ***Racial Ingroup > Outgroup*** |  |  |  |  |  |  |  |
| No Significant Clusters | n/a | n/a | n/a | n/a | n/a | n/a | n/a |
|  |  |  |  |  |  |  |  |
| ***Racial Outgroup > Ingroup*** |  |  |  |  |  |  |  |
| No Significant Clusters | n/a | n/a | n/a | n/a | n/a | n/a | n/a |
|  |  |  |  |  |  |  |  |

**Notes**. *Brodmann* = Brodmann area; *x, y, z* = coordinates in Montreal Neurological Institute (MNI) space; *k* = cluster size in mm^3^; *max* = maximum value within cluster; *mean* = average value within cluster. L = left, R = right. ^a^ = associated subclusters of RH Dorsal ACC. There were no significant Racial Ingroup > Outgroup or Racial Outgroup > Ingroup clusters for Social Categorization and Impression Formation tasks. All analyses were k-threshold corrected at *p<*.001.

**SM References**

Adams, R. B., Rule, N. O., Franklin, R. G., Wang, E., Stevenson, M. T., Yoshikawa, S., Nomura, M., et al. (2010). Cross-cultural reading the mind in the eyes: an fMRI investigation. *Journal of Cognitive Neuroscience*, *22*(1), 97–108.

Azevedo, R. T., Macaluso, E., Avenanti, A., Santangelo, V., Cazzato, V., & Aglioti, S. M. (2013). Their pain is not our pain: brain and autonomic correlates of empathic resonance with the pain of same and different race individuals. *Human Brain Mapping*, *34*(12), 3168–3181.

Berlingeri, M., Gallucci, M., Danelli, L., Forgiarini, M., Sberna, M., & Paulesu, E. (2016). Guess who’s coming to dinner: Brain signatures of racially biased and politically correct behaviors. *Neuroscience*, *332*, 231–241.

Bestelmeyer, P. E. G., Belin, P., & Ladd, D. R. (2015). A Neural Marker for Social Bias Toward In-group Accents. *Cerebral Cortex*, *25*(10), 3953–3961.

Brown, T. I., Uncapher, M. R., Chow, T. E., Eberhardt, J. L., & Wagner, A. D. (2017). Cognitive control, attention, and the other race effect in memory. *Plos One*, *12*(3), e0173579.

Cao, Y., Contreras-Huerta, L. S., McFadyen, J., & Cunnington, R. (2015). Racial bias in neural response to others’ pain is reduced with other-race contact. *Cortex*, *70*, 68–78.

Cheon, B. K., Im, D.-M., Harada, T., Kim, J.-S., Mathur, V. A., Scimeca, J. M., Parrish, T. B., et al. (2013). Cultural modulation of the neural correlates of emotional pain perception: the role of other-focusedness. *Neuropsychologia*, *51*(7), 1177–1186.

Chiao, J. Y., Iidaka, T., Gordon, H. L., Nogawa, J., Bar, M., Aminoff, E., Sadato, N., et al. (2008). Cultural specificity in amygdala response to fear faces. *Journal of Cognitive Neuroscience*, *20*(12), 2167–2174.

Contreras, J. M., Banaji, M. R., & Mitchell, J. P. (2013). Multivoxel patterns in fusiform face area differentiate faces by sex and race. *Plos One*, *8*(7), e69684.

Cunningham, W. A., Johnson, M. K., Raye, C. L., Chris Gatenby, J., Gore, J. C., & Banaji, M. R. (2004). Separable neural components in the processing of black and white faces. *Psychological Science*, *15*(12), 806–813.

Earls, H. A., Englander, Z. A., & Morris, J. P. (2013). Perception of race-related features modulates neural activity associated with action observation and imitation. *Neuroreport*, *24*(8), 410–413.

Falk, E. B., Spunt, R. P., & Lieberman, M. D. (2012). Ascribing beliefs to ingroup and outgroup political candidates: neural correlates of perspective-taking, issue importance and days until the election. *Philosophical Transactions of the Royal Society of London. Series B, Biological Sciences*, *367*(1589), 731–743.

Feng, L., Liu, J., Wang, Z., Li, J., Li, L., Ge, L., Tian, J., et al. (2011). The other face of the other-race effect: an fMRI investigation of the other-race face categorization advantage. *Neuropsychologia*, *49*(13), 3739–3749.

Freeman, J. B., Schiller, D., Rule, N. O., & Ambady, N. (2010). The neural origins of superficial and individuated judgments about ingroup and outgroup members. *Human Brain Mapping*, *31*(1), 150–159.

Hart, A. J., Whalen, P. J., Shin, L. M., McInerney, S. C., Fischer, H., & Rauch, S. L. (2000). Differential response in the human amygdala to racial outgroup vs ingroup face stimuli. *Neuroreport*, *11*(11), 2351–2355.

Hein, G., Silani, G., Preuschoff, K., Batson, C. D., & Singer, T. (2010). Neural responses to ingroup and outgroup members’ suffering predict individual differences in costly helping. *Neuron*, *68*(1), 149–160.

Junger, J., Pauly, K., Bröhr, S., Birkholz, P., Neuschaefer-Rube, C., Kohler, C., Schneider, F., et al. (2013). Sex matters: Neural correlates of voice gender perception. *Neuroimage*, *79*, 275–287.

Kaplan, J. T., Freedman, J., & Iacoboni, M. (2007). Us versus them: Political attitudes and party affiliation influence neural response to faces of presidential candidates. *Neuropsychologia*, *45*(1), 55–64.

Kober, H., Barrett, L. F., Joseph, J., Bliss-Moreau, E., Lindquist, K., & Wager, T. D. (2008). Functional grouping and cortical-subcortical interactions in emotion: a meta-analysis of neuroimaging studies. *Neuroimage*, *42*(2), 998–1031.

Kober, H., & Wager, T. D. (2010). Meta-analysis of neuroimaging data. *Wiley interdisciplinary reviews. Cognitive science*, *1*(2), 293–300.

Krautheim, J. T., Straube, B., Dannlowski, U., Pyka, M., Schneider-Hassloff, H., Drexler, R., Krug, A., et al. (2018). Outgroup emotion processing in the vACC is modulated by childhood trauma and CACNA1C risk variant. *Social Cognitive and Affective Neuroscience*, *13*(3), 341–348.

Krill, A., & Platek, S. M. (2009). In-group and out-group membership mediates anterior cingulate activation to social exclusion. *Frontiers in evolutionary neuroscience*, *1*, 1.

Lee, K.-U., Khang, H. S., Kim, K.-T., Kim, Y.-J., Kweon, Y.-S., Shin, Y.-W., Kwon, J. S., et al. (2008). Distinct processing of facial emotion of own-race versus other-race. *Neuroreport*, *19*(10), 1021–1025.

Li, T., Cardenas-Iniguez, C., Correll, J., & Cloutier, J. (2016). The impact of motivation on race-based impression formation. *Neuroimage*, *124*(Pt A), 1–7.

Li, X., Liu, Y., Luo, S., Wu, B., Wu, X., & Han, S. (2015). Mortality salience enhances racial in-group bias in empathic neural responses to others’ suffering. *Neuroimage*, *118*, 376–385.

Liu, Y., Lin, W., Xu, P., Zhang, D., & Luo, Y. (2015). Neural basis of disgust perception in racial prejudice. *Human Brain Mapping*, *36*(12), 5275–5286.

Losin, E. A. R., Cross, K. A., Iacoboni, M., & Dapretto, M. (2014). Neural processing of race during imitation: self-similarity versus social status. *Human Brain Mapping*, *35*(4), 1723–1739.

Losin, E. A. R., Iacoboni, M., Martin, A., Cross, K. A., & Dapretto, M. (2012). Race modulates neural activity during imitation. *Neuroimage*, *59*(4), 3594–3603.

Losin, E. A. R., Iacoboni, M., Martin, A., & Dapretto, M. (2012). Own-gender imitation activates the brain’s reward circuitry. *Social Cognitive and Affective Neuroscience*, *7*(7), 804–810.

Luo, S., Li, B., Ma, Y., Zhang, W., Rao, Y., & Han, S. (2015). Oxytocin receptor gene and racial ingroup bias in empathy-related brain activity. *Neuroimage*, *110*, 22–31.

Mathur, V. A., Harada, T., Lipke, T., & Chiao, J. Y. (2010). Neural basis of extraordinary empathy and altruistic motivation. *Neuroimage*, *51*(4), 1468–1475.

Mattan, B. D., Kubota, J. T., Dang, T. P., & Cloutier, J. (2018). External motivation to avoid prejudice alters neural responses to targets varying in race and status. *Social Cognitive and Affective Neuroscience*, *13*(1), 22–31.

Molenberghs, P., Bosworth, R., Nott, Z., Louis, W. R., Smith, J. R., Amiot, C. E., Vohs, K. D., et al. (2014). The influence of group membership and individual differences in psychopathy and perspective taking on neural responses when punishing and rewarding others. *Human Brain Mapping*, *35*(10), 4989–4999.

Molenberghs, P., Gapp, J., Wang, B., Louis, W. R., & Decety, J. (2016). Increased moral sensitivity for outgroup perpetrators harming ingroup members. *Cerebral Cortex*, *26*(1), 225–233.

Molenberghs, P., & Morrison, S. (2014). The role of the medial prefrontal cortex in social categorization. *Social Cognitive and Affective Neuroscience*, *9*(3), 292–296.

Molenberghs, P., Prochilo, G., Steffens, N. K., Zacher, H., & Haslam, S. A. (2017). The Neuroscience of Inspirational Leadership: The Importance of Collective-Oriented Language and Shared Group Membership. *Journal of management*, *43*(7), 2168–2194.

Morrison, S., Decety, J., & Molenberghs, P. (2012). The neuroscience of group membership. *Neuropsychologia*, *50*(8), 2114–2120.

Rauchbauer, B., Majdandžić, J., Hummer, A., Windischberger, C., & Lamm, C. (2015). Distinct neural processes are engaged in the modulation of mimicry by social group-membership and emotional expressions. *Cortex*, *70*, 49–67.

Richeson, J. A., Baird, A. A., Gordon, H. L., Heatherton, T. F., Wyland, C. L., Trawalter, S., & Shelton, J. N. (2003). An fMRI investigation of the impact of interracial contact on executive function. *Nature Neuroscience*, *6*(12), 1323–1328.

Richeson, J. A., Todd, A. R., Trawalter, S., & Baird, A. A. (2008). Eye-Gaze Direction Modulates Race-Related Amygdala Activity. *Group Processes & Intergroup Relations*, *11*(2), 233–246.

Richins, M. T., Barreto, M., Karl, A., & Lawrence, N. (2019). Empathic responses are reduced to competitive but not non-competitive outgroups. *Social Neuroscience*, *14*(3), 345–358.

Ronquillo, J., Denson, T. F., Lickel, B., Lu, Z.-L., Nandy, A., & Maddox, K. B. (2007). The effects of skin tone on race-related amygdala activity: an fMRI investigation. *Social Cognitive and Affective Neuroscience*, *2*(1), 39–44.

Ruckmann, J., Bodden, M., Jansen, A., Kircher, T., Dodel, R., & Rief, W. (2015). How pain empathy depends on ingroup/outgroup decisions: A functional magnet resonance imaging study. *Psychiatry Research*, *234*(1), 57–65.

Rule, N. O., Freeman, J. B., Moran, J. M., Gabrieli, J. D. E., Adams, R. B., & Ambady, N. (2010). Voting behavior is reflected in amygdala response across cultures. *Social Cognitive and Affective Neuroscience*, *5*(2–3), 349–355.

Sheng, F., Liu, Q., Li, H., Fang, F., & Han, S. (2014). Task modulations of racial bias in neural responses to others’ suffering. *Neuroimage*, *88*, 263–270.

Telzer, E. H., Ichien, N., & Qu, Y. (2015). The ties that bind: Group membership shapes the neural correlates of in-group favoritism. *Neuroimage*, *115*, 42–51.

Van Bavel, J. J., Packer, D. J., & Cunningham, W. A. (2008). The neural substrates of in-group bias: a functional magnetic resonance imaging investigation. *Psychological Science*, *19*(11), 1131–1139.

Volz, K. G., Kessler, T., & von Cramon, D. Y. (2009). In-group as part of the self: In-group favoritism is mediated by medial prefrontal cortex activation. *Social Neuroscience*, *4*(3), 244–260.

Wager, T. D., Lindquist, M., & Kaplan, L. (2007). Meta-analysis of functional neuroimaging data: current and future directions. *Social Cognitive and Affective Neuroscience*, *2*(2), 150–158.

Watson, R., & de Gelder, B. (2017). How white and black bodies are perceived depends on what emotion is expressed. *Scientific Reports*, *7*, 41349.

Wheeler, M. E., & Fiske, S. T. (2005). Controlling racial prejudice: social-cognitive goals affect amygdala and stereotype activation. *Psychological Science*, *16*(1), 56–63.

Wu, C.-T., Fan, Y.-T., Du, Y.-R., Yang, T.-T., Liu, H.-L., Yen, N.-S., Chen, S.-H., et al. (2018). How Do Acquired Political Identities Influence Our Neural Processing toward Others within the Context of a Trust Game? *Frontiers in Human Neuroscience*, *12*, 23.

Xu, X., Zuo, X., Wang, X., & Han, S. (2009). Do you feel my pain? Racial group membership modulates empathic neural responses. *The Journal of Neuroscience*, *29*(26), 8525–8529.
